# Hydroxyurea affects *in vitro* porcine oocyte maturation through increased apoptosis and oxidative stress

**DOI:** 10.1042/BSR20203091

**Published:** 2021-04-22

**Authors:** Wei Gao, Yongxun Jin, Jindong Hao, Siyi Huang, Dongxu Wang, Fushi Quan, Mingjun Zhang, Jiabao Zhang, Wenzhi Ren, Xianfeng Yu

**Affiliations:** Jilin Provincial Key Laboratory of Animal Model, College of Animal Science, Jilin University, Changchun 130062, China

**Keywords:** Apoptosis, Hydroxyurea, Maturation, Oxidative stress, Porcine oocyte

## Abstract

Hydroxyurea (HU) is an FDA-approved drug used to treat a variety of diseases, especially malignancies, but is harmful to fertility. We used porcine oocytes as an experimental model to study the effect of HU during oocyte maturation. Exposure of cumulus–oocyte complexes (COCs) to 20 µM (*P*<0.01) and 50 µM (*P*<0.001) HU reduced oocyte maturation. Exposure to 20 µM HU induced approximately 1.5- and 2-fold increases in Caspase-3 (*P*<0.001) and P53 (*P*<0.01) gene expression levels in cumulus cells, respectively, increased Caspase-3 (*P*<0.01) and P53 (*P*<0.001) protein expression levels in metaphase II (MII) oocytes and increased the percentage of apoptotic cumulus cells (*P*<0.001). In addition, HU decreased the mitochondrial membrane potential (Δφm) (*P*<0.01 and *P*<0.001) and glutathione (GSH) levels (*P*<0.01 and *P*<0.001) of both cumulus cells and MII oocytes, while increasing their reactive oxygen species (ROS) levels (*P*<0.001). Following parthenogenetic activation of embryos derived from MII oocytes, exposure to 20 µM HU significantly reduced total blastocyst cell numbers (*P*<0.001) and increased apoptosis of blastocyst cells (*P*<0.001). Moreover, HU exposure reduced the rate of development of two-celled, four- to eight-celled, blastocyst, and hatching stages after parthenogenetic activation (*P*<0.05). Our findings indicate that exposure to 20 µM HU caused significant oxidative stress and apoptosis of MII oocytes during maturation, which affected their developmental ability. These results provide valuable information for safety assessments of HU.

## Introduction

Hydroxyurea (HU), a United States Food and Drug Administration-approved drug [1], is an inhibitor of ribonucleotide reductase commonly used to treat myeloproliferative disorders and sickle cell anemia [2]. In addition, HU is used as an anti-tumor drug to treat various malignancies [3]. However, HU has several adverse effects and should be used with caution in pregnant women and children. HU can cause abnormal embryonic development in mice, rats, and New Zealand white rabbits [4–9]. Moreover, studies have shown that use of HU in pregnant women or babies can cause harmful effects [10,11]. In addition, HU can be extremely toxic to preimplantation embryos because it impacts blastocyst formation and development, compromises folliculogenesis, and reduces ovulation [12]. HU inactivates ribonucleotide reductase and inhibits DNA synthesis in proliferating cells, and can increase apoptosis and induce cell cycle changes [11,13,14]. Accordingly, HU exposure induced apoptosis of fetal tissue cells, which resulted in abnormal tissue development in offspring [15]. HU can increase the production of reactive oxygen species (ROS) [8,16]. The carbamoyl nitroso group is an intermediate of HU that can participate in electron transfer, ROS formation, and oxidative stress [17]. As HU compromises folliculogenesis and can elicit apoptosis and oxidative stress, we hypothesized that HU causes apoptosis and oxidative stress during oocyte maturation. Most research on HU has focused on effects during pre-implantation and post-implantation embryo development, while few reports describe the effects of HU during oocyte maturation. To address this need, the current study investigated the effects of HU on apoptosis and oxidative stress during maturation of porcine oocytes.

## Materials and methods

### Reagents

All chemicals and reagents, except those specifically noted, were purchased from Sigma–Aldrich (St. Louis, MO, U.S.A.).

### Drug treatment and experimental design

Previous studies exposed embryos to 2 mM HU for 2 h [18] or 0.237 mM for 1 h [12]. Therefore, we used *in vitro* maturation (IVM) medium supplemented with 1, 10, 20, or 50 µM HU for 46–48 h.

IVM media supplemented with different concentrations of HU (0, 1, 10, 20, or 50 µM) were prepared prior to incubation with oocytes to examine maturation. We analyzed numbers of metaphase II (MII) oocytes, as well as glutathione (GSH) and ROS levels, Δφm, and apoptosis.

Next, we cultured parthenogenetically activated MII oocytes [matured in IVM medium with 0 or 20 µM HU, followed by *in vitro* culture (IVC) medium without HU] to test the effect of HU on development of MII oocytes to blastocysts. We analyzed rates of blastocyst formation, total blastocyst cell numbers, and apoptosis of blastocyst cells.

Finally, we cultured parthenogenetically activated MII oocytes (matured in IVM medium without HU) in IVC medium containing 0, 1, 10, 20, or 50 µM HU to test the effect of HU on embryo development.

### Collection of porcine oocytes and IVM

Approximately 200 porcine ovaries (obtained from the slaughterhouse) were stored in warm 0.9% saline solution containing 1% antibiotic and sent to the laboratory within 3 h. Using sterile syringes, porcine cumulus–oocyte complexes (COCs) were extracted from 3–6 mm diameter follicles and placed in a 50-ml tube. Collected COCs were washed three times by adding Tyrode’s lactate-HEPES buffered medium containing 1% antibiotic and 1 g/l polyvinyl alcohol (PVA). Next, 40–60 COCs were added to 450 μl of IVM medium (TCM-199; 0.91 mM sodium pyruvate, 75 mg/ml kanamycin, 0.6 mM l-cysteine, 10 ng/ml epidermal growth factor, 10 IU/ml luteinizing hormone, 10 IU/ml follicle-stimulating hormone, and 10% v/v porcine follicular fluid) per well of non-tissue culture-treated four-well plates (179830, Thermo Scientific, Waltham, MA, U.S.A.) and covered with 500 μl mineral oil. COCs were cultured in IVM medium for 44–46 h at 38.5°C with 5% CO_2_/95% air. After maturation, we used HEPES buffer containing 1 mg/ml hyaluronidase to remove cumulus cells from COCs and collected cumulus cells and MII oocytes. We selected MII oocytes using a stereo microscope (180–400× magnification) with a heating stage at 38.5°C. A 200-μm-diameter glass needle was used to collect oocytes with uniform cytoplasm and an extruded first polar body. We analyzed the rate of MII oocyte development at 46 h.

### Parthenogenetic activation and IVC

The parthenogenetic activation system employed followed our previously described method [19,20]. Isolated MII oocytes (without cumulus cells) exhibiting homogeneous cytoplasm were used for activation. MII oocytes were incubated in activation medium (280 mM mannitol, 0.01 mM CaCl_2_, and 0.05 mM MgCl_2_) for 2 min and then placed into an activation slot for activation with electrical pulses (1.0 kV/cm for 60 ms). Next, 40–60 activated MII oocytes/well were incubated in activation PZM-5 medium [7.5 μg/ml cytochalasin B, 0.4 mM MgSO_4_.7H_2_0, 108 mM NaCl, 2.0 mM l-glutamine, 20 ml/l BME amino acids, 10 ml/l MEM non-essential amino acids, 10 mM KCl, 0.35 mM KH_2_PO_4_, 5.0 mM hypotaurine, 25.07 mM NaHCO_3_, 0.2 mM Na pyruvate, 2.0 mM Ca-(lactate)_2_.5H_2_0, 25 mg/ml gentamycin, 4 mg/ml bovine serum albumin (BSA), 28.516 μM l-cysteine]. After 4 h, activated MII oocytes were transferred into 450 μl IVC medium and covered with 500 μl mineral oil, and culture dishes were placed in an embryo incubator at 38.5°C with 5% CO_2_ and 95% humidity. Two-celled, four- to eight-celled, blastocyst, and hatching rates were analyzed at 46, 46–72, 168, and 192 h.

### Evaluation of total cell numbers per blastocyst

Total blastocyst cell numbers were determined for 21 control group blastocysts (7 per group, matured in IVM medium followed by IVC medium without HU) and 21 HU-exposed blastocysts (7 per group, matured in IVM medium with 20 µM HU, followed by IVC medium without HU). Blastocysts were fixed with 4% (w/v) paraformaldehyde, washed three times with phosphate-buffered saline mixed with 1g/l PVA (PBS-PVA), and incubated in 10 μg/ml Hoechst 33342 for 5 min at 37°C. Finally, blastocysts were placed on a glass slide under a glass coverslip, and images were acquired with a digital camera and fluorescence microscope (E179168, Nikon, Tokyo, Japan).

### Immunofluorescence staining and real-time reverse transcription polymerase chain reaction

MII oocytes were washed three times with PBS-PVA, fixed with 4% (w/v) paraformaldehyde solution, washed, and incubated with 0.2% (v/v) Triton X-100 for 15–20 min. Fixed oocytes were washed and incubated in 1% (w/v) BSA for 1 h at room temperature to block nonspecific binding. Oocytes were incubated with anti-P53 (1:100; Abcam, Cambridge, U.K.) and anti-Caspase-3 (1:100; Abcam) antibodies at 4°C overnight. The following day, oocytes were washed, incubated with a secondary antibody (1:100; CY3-goat anti-rabbit; Boster Biological Technology, Wuhan, China) at 37°C for 1–2 h, washed three times, and placed in Hoechst 33342 for 5 min at 37°C. We used 24 (8 per group, control group) and 24 (8 per group, 20 μM HU group) MII oocytes to examine P53 expression, and 21 (7 per group, control group) and 21 (7 per group, 20 μM HU group) MII oocytes to examine Caspase-3 expression. Immunostained oocytes were placed on glass slides and covered with a glass coverslip. Images were acquired with a digital camera with a fluorescence microscope.

Total mRNA was extracted from 5 × 10^4^ digested cumulus cells (from 20 MII oocytes) using a microRNA extraction kit (Qiagen, Dusseldorf, Germany). mRNA was reverse transcribed into cDNA using a reverse transcription kit (Tiangen Biotech, Beijing, China). SYBR green fluorescent dye (Tiangen Biotech), cDNA, ddH_2_O, and primers (Supplementary Table S1) were added to the samples for PCR using a reverse transcription polymerase chain reaction (RT-PCR) instrument (Eppendorf, Hamburg, Germany). RT-PCR cycles included pre-denaturation at 95°C for 15 min followed by 45 cycles of 95°C for 10 s (denaturation), 60°C for 20 s (annealing), and 72°C for 30 s (extension), followed by melting curve analysis. The *β*-Actin gene was used for standardization. Three independent experiments were performed, and the 2^−ΔΔ*C*_t_^ [ΔΔ*C*_t_ = Δ*C*_t_ (case) − Δ*C*_t_ (control)] method was used to calculate relative mRNA expression.

### Flow cytometry detection of apoptosis in cumulus cells

Digested cumulus cells were collected using a low-speed centrifuge and washed in a 1.5-ml tube. The PBS supernatant was gently decanted after centrifugation. PBS-PVA cleaning solution was added and the cell slurry was gently mixed by pipetting. Following three washes, a solution containing 5 µl of Annexin V-FITC (Ca^2+^-dependent phospholipid binding protein; Solarbio Life Sciences, Beijing, China) was added to samples, which were mixed at 20°C for 10 min. Next, 5 µl of propidium iodide (Solarbio Life Sciences) was added to samples for incubation at 20°C for 5 min. Finally, samples were gently mixed with 500 µl of PBS (all steps in the dark) and analyzed within 1 h by flow cytometry.

### TUNEL assay for detection of apoptosis in blastocysts

To measure apoptosis levels in blastocysts, 18 (6 per group) and 18 (6 per group) blastocysts from parthenogenetically activated MII oocytes (matured in IVM medium with 0 or 20 µM HU, followed by culture in IVC medium without HU) were washed three times with PBS-PVA, fixed with 4% (w/v) paraformaldehyde solution, washed, and incubated with 0.2% (v/v) Triton X-100 for 15–20 min. After washing three times with PBS-PVA, fixed blastocysts were incubated with TdT and fluorescein-conjugated dUTPs (In Situ Cell Death Detection Kit; Roche, Mannheim, Germany) in the dark for 30 min at 37°C. Next, blastocysts were washed three times, placed in Hoechst 33342 for 5 min at 37°C, and washed again three times with PBS-PVA for 10 min each. Immunostained blastocysts were placed on glass slides and covered with glass coverslips. Images were acquired with a digital camera and fluorescence microscope.

### GSH and ROS levels in MII oocytes and cumulus cells

To measure GSH levels, 48 (15–17 per group) and 50 (16–18 per group) MII oocytes from control and 20 μM HU groups, respectively, were incubated with IVM medium containing 10 µM of 4-chloromethyl-6,8-difluoro-7-hydroxycoumarin (CMF2HC, Thermo Fisher Scientific) cell tracer blue dye for 20 min, washed three times, and analyzed by spectroscopy (blue fluorescence, UV filter, 370 nm). To examine ROS levels, 48 (15–17 per group) MII oocytes from control and 20 μM HU groups were washed three times with PBS-PVA and incubated with IVM medium containing 10 µM of 2,7-dichlorodihydrofluorescein diacetate (H2DCFDA; Thermo Fisher Scientific) for 15 min in non-treated four-well plates. Following incubation, plates were washed three times and analyzed using a fluorescence microscope (green fluorescence, UV filters, 490 nm). All images were stored as TIFF files and analyzed by ImageJ software (http://imagej.nih.gov).

Cumulus cells were washed three times for 5 min at 600×***g*** in 1.5-ml microcentrifuge tubes. Next, cells were incubated with IVM medium containing 10 µM CMF2HC or 10 µM H2DCFDA for 20 min and 15 min, respectively, followed by three washes. Cumulus cells incubated with CMF2HC were analyzed by a fluorescence microscope (blue fluorescence, UV filter, 370 nm). Images were stored as TIFF files and analyzed by ImageJ software. Approximately 1–5 × 10^4^ cumulus cells incubated with H2DCFDA were placed on ice in the dark for analysis by flow cytometry (green fluorescence, UV filters, 490 nm). Expression of 2′,7′-dichlorofluorescein is presented as the peak of the median FITC-A signal.

### Δφm of MII oocytes and cumulus cells

Δφm was calculated as the ratio of red fluorescence (corresponding to activated mitochondria) to green fluorescence (corresponding to less activated mitochondria, J-monomer). Forty-five (15 per group) MII oocytes from control and 20 μM HU groups were washed three times with PBS-PVA, then incubated with IVM medium containing JC-1 fluorescent probe (Solarbio Life Sciences) at 37°C for 20 min. After washing three times with JC-1 staining buffer, 15 oocytes per well were placed in 15 μl of JC-1 staining buffer and covered with 50 μl of mineral oil in six-well plates.

For measurement of Δφm, cumulus cells were washed three times for 5 min at 600×***g*** in 1.5-ml microcentrifuge tubes. Next, cells were incubated with IVM medium containing JC-1 fluorescent probe at 37°C for 20 min, and then washed three times with JC-1 staining buffer. Cells were placed in 1 ml of JC-1 staining buffer per tube and transferred in 200-μl aliquots to wells of a six-well plate for analysis. Cells were analyzed using a fluorescence microscope with 490-nm (green fluorescence) and 530-nm (red fluorescence) excitation. Images stored as TIFF files were analyzed by ImageJ software.

### Statistical analysis

Each experiment was repeated at least three times and data were analyzed by SPSS 20.0 software (IBM, Armonk, NY, U.S.A.). We used Student’s *t* test to analyze comparisons of two groups and ANOVA test to analyze comparisons of more than two groups. *P*<0.05 was considered statistically significant.

## Results

### Effect of 1, 10, 20, and 50 µM HU on IVM and IVC of porcine oocytes

**Figure 1:**
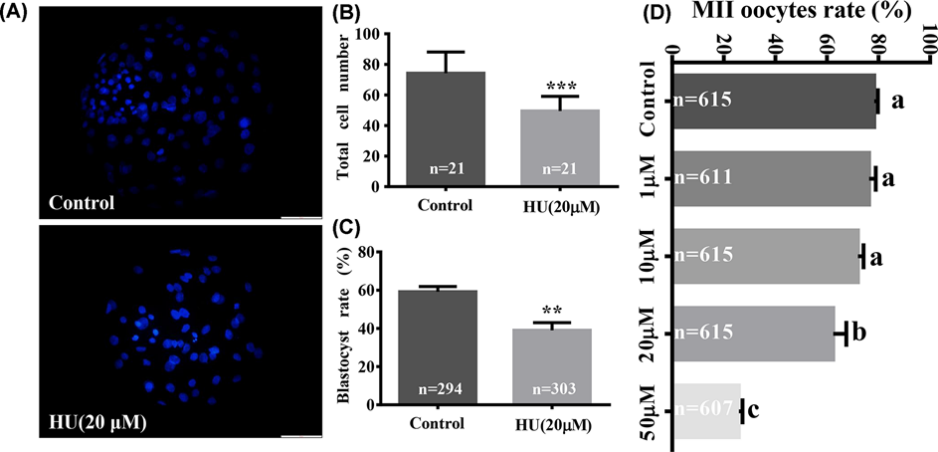
Effects of HU on porcine oocyte maturation rate, blastocyst rate, and total cell numbers for parthenogenetically activated embryos Cumulus oocyte complexes were cultured in IVM media without (control) or with 1, 10, 20, or 50 µM HU. The resulting MII oocytes were cultured in IVC media without HU. (**A**) Images of Hoechst 33342-stained cells from control and 20-µM HU-exposed groups. Scale bar = 50 μm. (**B**) Histogram of blastocyst total cell numbers for control and 20 µM HU-exposed groups. (**C**) Maturation rates to blastocyst stage for control and 20-µM HUexposed groups. (**D**) Shows the MII oocyte maturation rate of control and 1-, 10-, 20-, or 50-µM HU-exposed groups. Values shown are mean ± standard deviation of three independent experiments. **P<0.01, and ***P<0.001. a, b, and c show the sorting of averages; a is the largest average, *P*<0.05 indicates a significant difference between the two groups.

Porcine COCs were cultured in IVM media supplemented with 1, 10, 20, or 50 µM HU to determine the effect of HU on MII oocyte development. Exposure to 20 or 50 µM HU significantly decreased the percentage of MII oocytes compared with the control group (62.43 ± 5.13% and 25.86 ± 1.44% vs. 78.35 ± 1.43%, respectively, Figure 1D). Therefore, 20 and 50 µM HU affected polar body extrusion in porcine oocytes.

MII oocytes cultured in IVM media with (20 µM) or without HU were subsequently cultured in IVC media to examine post-meiotic development to the blastocyst stage. Percentages of blastocysts were significantly decreased in the 20-µM HU group compared with the control group (39.00 ± 4.04% vs. 59.17 ± 2.78%, *P*<0.01, Figure 1C). Moreover, total blastocyst cell numbers were significantly decreased in the 20-µM HU group compared with the control group (49.52 ± 9.55 vs. 74.19 ± 13.92, Figure 1A,B, *P*<0.001). These results indicate that 20 µM HU affected post-meiotic development of MII oocytes.

**Figure 2:**
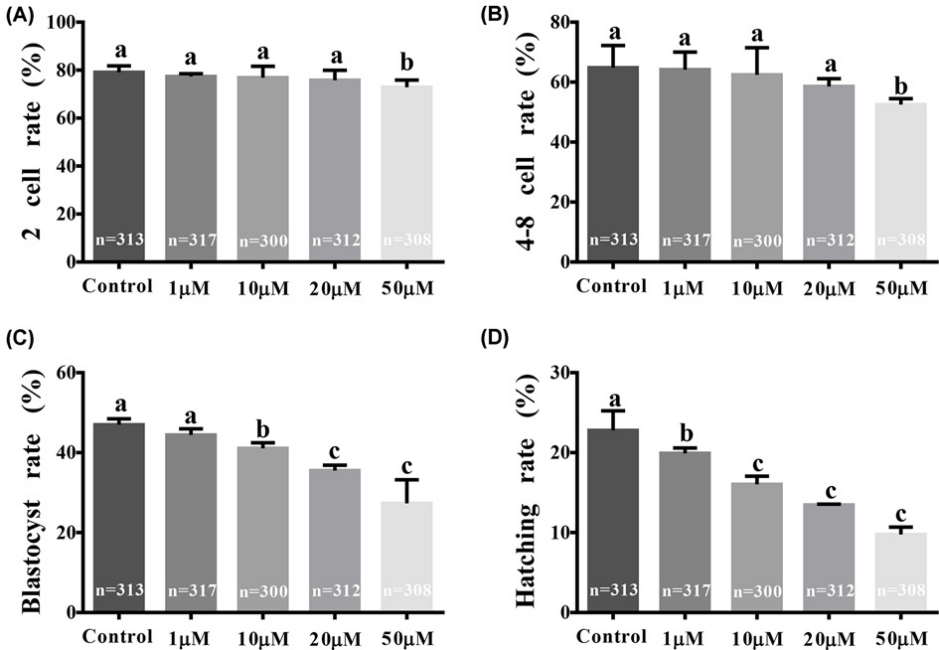
Effect of HU on embryonic development after parthenogenic activation of pig embryos Cumulus oocyte complexes were cultured in IVM media without HU, and the resulting MII oocytes were incubated in IVC media supplemented with 0, 1, 10, 20 or 50 µM HU. (**A**) Rates of maturation to the two-celled stage. (**B**) Rates of maturation to the four- to eight-celled stage. (**C**) Maturation rates to the blastocyst stage. (**D**) Hatching rates. Bars represent values of three independent replicate experiments. Values are mean ± standard deviation. a, b, c show the sorting of averages; a is the largest average, *P*<0.05 indicates a significant difference between the two groups.

Porcine COCs were cultured in IVM media without HU, and the resulting MII oocytes were cultured in IVC media supplemented with 0, 1, 10, 20, or 50 µM HU to examine the effect of HU on two-celled to hatching stage embryonic development. Percentages of two- and four- to eight-celled embryos were significantly decreased in the 50-µM HU group compared with the control group (72.89 ± 2.99% vs. 79.15 ± 2.65%, *P*<0.05, Figure 2A; and 52.52 ± 1.95% vs. 64.80 ± 7.45%, *P*<0.05, Figure 2B, respectively). Blastocyst formation rates of 10-, 20-, and 50-µM HU groups were significantly decreased compared with the control group [41.07 ± 1.41% (*P*<0.05), 35.55 ± 1.33% (*P*<0.01), and 27.33 ± 5.90% (*P*<0.001) vs. 46.96 ± 1.53%, respectively, Figure 2C]. Hatching rates in the 1-, 10-, 20-, and 50-µM HU groups were significantly decreased compared with the control group [19.92 ± 0.66% (*P*<0.001), 16.04 ± 1.02% (*P*<0.001), 13.46 ± 0.11% (*P*<0.001), and 9.77 ± 0.93% (*P*<0.001) vs. 22.79 ± 2.45%, respectively, Figure 2D]. These results indicate that 1, 10, 20, and 50 µM HU affected MII oocyte development during the two-celled to hatching stage.

### Apoptosis of MII oocytes, cumulus cells, and parthenogenetically activated blastocysts exposed to 20 µM HU

**Figure 3:**
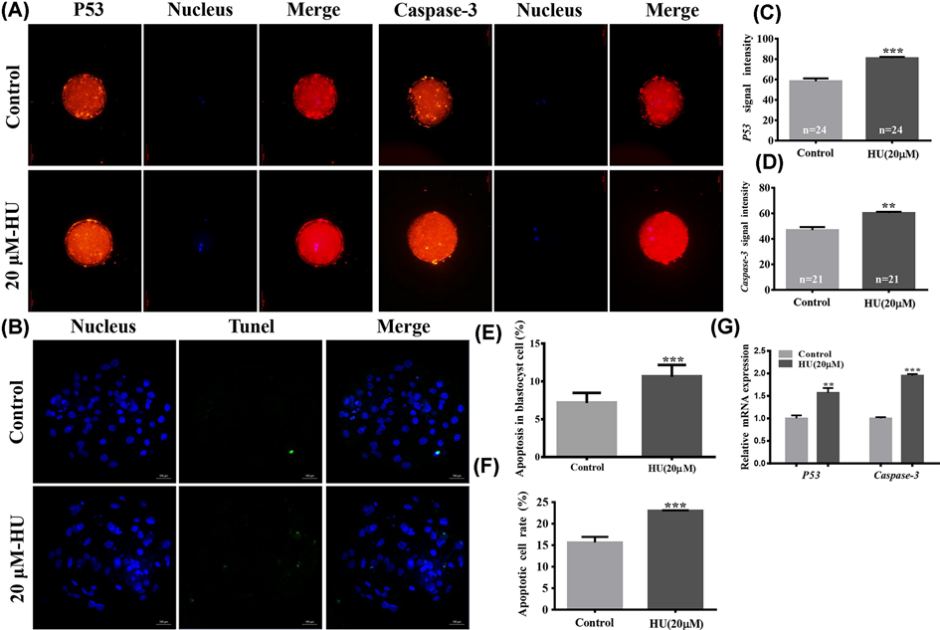
Apoptosis levels of MII oocytes, cumulus cells, and blastocysts exposed to HU Cells were cultured in IVM media supplemented with 0 or 20 µM HU. (**A**) *P53* and Caspase-3 levels in MII oocytes, with apoptotic proteins labeled with red fluorescence and blue indicating nuclei. Scale bar = 100 μm.(**B**) dUTPs labeled with green fluorescence and blue, indicating nuclei, in blastocysts. Scale bar = 100 μm. (**C**) Signal strength of *P53* protein expression. (**D**) Signal strength of Caspase-3 protein expression. (**E**) Percentage of apoptotic cells in blastocysts. (**F**) Percentage of apoptotic cumulus cells. (**G**) Relative expression levels of P53 and Caspase-3 mRNA in cumulus cells. Values indicate mean ± standard deviation of three independent experiments. **P<0.01, ***P<0.001.

To determine the effect of HU on apoptosis, P53 and Caspase-3 expression levels were evaluated using immunofluorescence staining. Expression of P53 protein in MII oocytes was increased in the 20-µM HU group compared with the control group (80.84 ± 1.23 vs. 58.16 ± 2.90 pixels per oocyte, *P*<0.001, Figure 3A,C, respectively). Expression of Caspase-3 protein in MII oocytes was also increased following HU exposure compared with the control group (60.16 ± 1.04 vs. 46.82 ± 2.41 pixels per oocyte, *P*<0.01, Figure 3A,D, respectively).

Analysis of cumulus cell mRNA showed increased levels of Caspase-3 (1.95 ± 0.04 vs. 1.00 ± 0.03, *P*<0.001, Figure 3G) and P53 (1.57 ± 0. 11 vs. 1.00 ± 0.07, *P*<0.01, Figure 3G) expression following 20 µM HU exposure. In addition, flow cytometry analyses showed increased apoptosis in 20-µM-HU-exposed cumulus cells (23.03 ± 0.07% vs. 17.13 ± 0.21, *P*<0.001, Figure 3F). In parthenogenetically activated blastocysts derived from MII oocytes (matured in IVM medium with 20 µM HU), the percentage of apoptotic cells (10.60 ± 1.57 vs. 7.13 ± 1.34, *P*<0.001, Figure 3B,E) was significantly increased.

### GSH and ROS levels in MII oocytes and cumulus cells exposed to 20 µM HU

**Figure 4:**
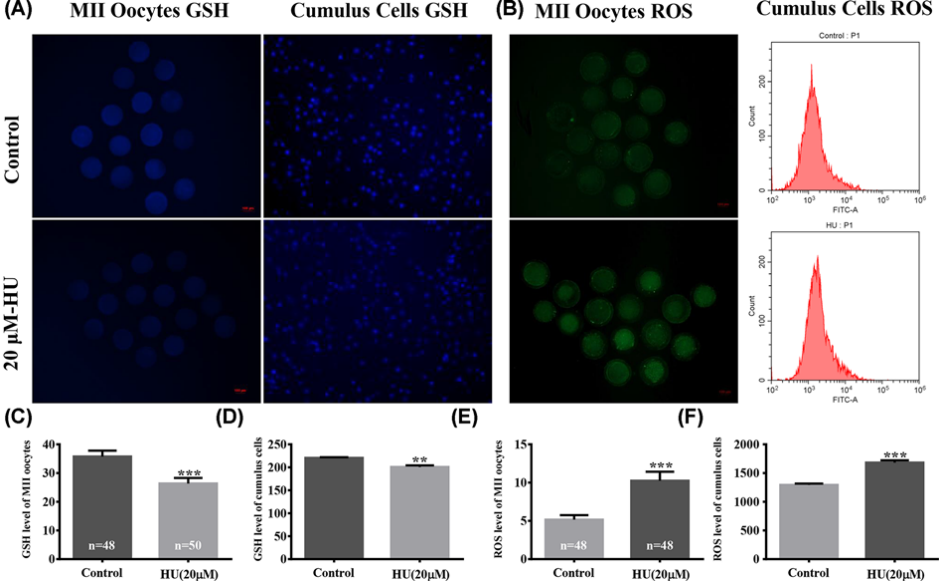
Effect of HU on intracellular GSH and ROS levels in MII oocytes and cumulus cells Cells were cultured in IVM media supplemented with 0 or 20 µM HU. (**A**) Intracellular CMF2HC-stained (GSH) MII oocytes and cumulus cells following exposure to 0 or 20 µM HU (scale bar = 100 μm). (**B**) Intracellular H2DCFDA-stained (ROS) MII oocytes following exposure to 0 or 20 µM HU (scale bar = 100 μm), and median FITC-A values of H2DCFDA-treated cumulus cells by flow cytometry. (**C,D**) GSH signal intensity of MII oocytes and cumulus cells. (**E,F**) indicate the ROS signal intensity of MII oocytes and cumulus cells. The experiment was repeated three times and values shown are mean ± standard deviation. **P<0.01, and ***P<0.001.

To understand the mechanism of action by which HU affected porcine oocyte maturation, GSH and ROS levels were measured after *in vitro* oocyte maturation. ROS levels in MII oocytes were significantly higher in the 20-µM-HU-exposed group versus the control group (10.24 ± 1.19 vs. 5.15 ± 0.59 pixels per oocyte, *P*<0.001, Figure 4B,E). Conversely, GSH was significantly down-regulated in the 20-µM HU group (26.35 ± 1.95 vs. 35.75 ± 2.04 pixels per oocyte, *P*<0.001, Figure 4A,C).

In parallel with fluorometric measurements, fluorescence microscopy measurements showed decreased levels of GSH (200.66 ± 3.48 vs. 220.45 ± 1.53, *P*<0.01, Figure 4A,D) in cumulus cells exposed to 20 µM HU. In addition, flow cytometry was used to measure ROS levels in cumulus cells. Cumulus cells exposed to 20 µM HU exhibited a significant increase in ROS (1681.25 ± 40.75 vs. 1292.35 ± 23.65, *P*<0.001, Figure 4B,F).

### Δφm of MII oocytes and cumulus cells exposed to 20 µM HU

**Figure 5:**
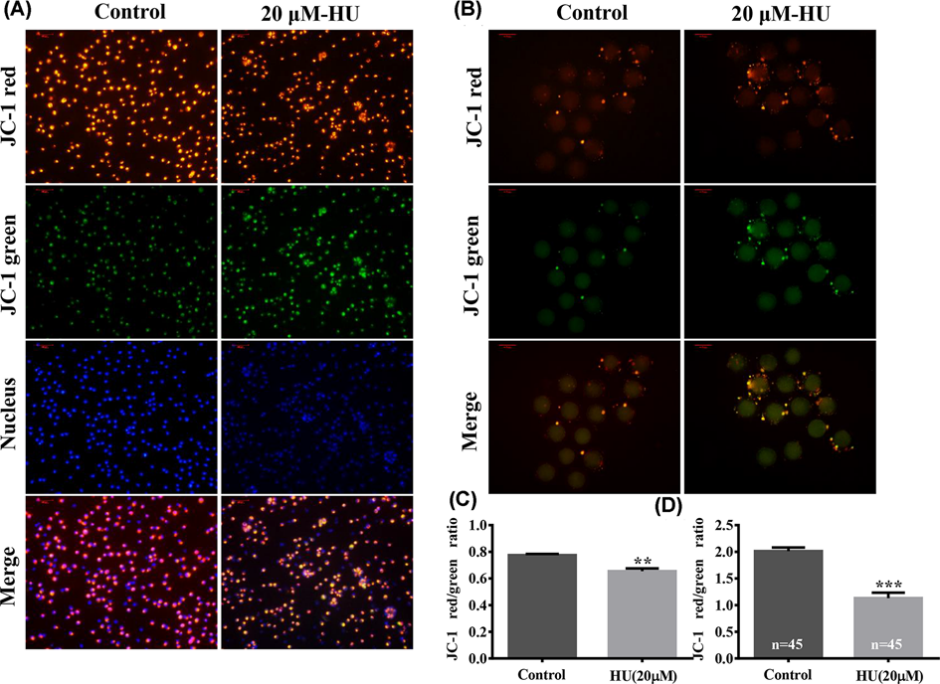
Effect of HU on Δφm of MII oocytes and cumulus cells Cells were cultured in IVM media supplemented with 0 or 20 µM HU. (**A,B**) Show staining of intracellular JC-1 aggregates (red) and JC-1 monomers (green) in cumulus cells and MII oocytes after exposure to 0 or 20 µM HU (scale bar = 100 μm). (**C,D**) Show the ratio of red fluorescence to green fluorescence. Bars represent values of three independent replicate experiments. Values shown are mean ± standard deviation. **P<0.01 and ***P<0.001.

Additional investigation into the mechanism of action of HU focused on impacts to the Δφm of MII oocytes and cumulus cells. Exposure to 20 µM HU significantly reduced Δφm (0.65 ± 0.02 vs. 0.77 ± 0.01, *P*<0.01, Figure 5A,C) in cumulus cells and in oocytes (1.13 ± 0.10 vs. 2.01 ± 0.07, *P*<0.001, Figure 5B,D).

## Discussion

Our results show that HU decreased the maturation rate of MII oocytes and significantly decreased the parthenogenetic activation rate of blastocysts. In addition, HU significantly decreased total blastocyst cell numbers. These findings indicate that HU can decrease the maturation and developmental ability of porcine oocytes. We also investigated the effects of HU on the two-celled to hatching stage of porcine embryonic development and found that HU significantly reduced development during the two-celled to hatching stage. Our experimental results are consistent with previous studies [12,21] showing that HU inhibits early embryonic development.

The P53 pathway plays a central role in embryonic stress response and teratogenesis [22]. While activation of the P53 pathway in aneuploid cells reduces cell proliferation [23], P53 activation leads to cell cycle arrest and apoptosis during embryonic kidney development and in mouse embryonic stem cells [24,25]. During the organogenesis stage of embryonic development, HU significantly increased levels of P53 and the P53-dependent protein Caspase-3 [26]. In the current study, we observed a similar increase in both P53 and Caspase-3 protein levels following HU exposure of MII oocytes. The observed effects in oocytes demonstrate HU-induced toxicity in post-implantation embryos. Past work showed that HU induced high levels of P53 expression during IVM of oocytes, which led to cell developmental arrest and disrupted extrusion of the second polar body [21]. Therefore, HU exposure results in reduced maturity of oocytes, affecting their IVM. During maturation, signaling between cumulus cells and oocytes elicits interactions of cell-secreted factors [27,28]. Proliferation and differentiation of cumulus cells are essential for the development of oocytes [29–33]. The maturation of cumulus cells greatly influences the maturation of porcine oocytes. Previous work showed that reduced apoptosis of porcine cumulus cells leads to decreased expression of Caspase-3 and related apoptotic proteins, as well as a reduced BCL2/BAX ratio [34]. Additionally, increased expression levels of apoptotic genes, such as p38, have been observed during porcine maturation [35,36]. We observed increased levels of P53 and Caspase-3 mRNA following HU exposure of cumulus cells. In parallel, flow cytometry and TUNEL assay analyses showed increased cumulus and blastocyst cell apoptosis because of HU exposure. Our results confirm that apoptosis of cumulus cells and oocytes is synchronized, but the mechanisms of HU-mediated apoptosis of cumulus cells and oocytes remain undetermined and need further study. We hypothesized that HU blocks oocyte maturation by increasing apoptosis of both oocytes and cumulus cells, and by directly inhibiting oocyte maturation.

During embryonic development, HU has been shown to induce nitric oxide (NO) through regulation of production and binding to fetal hemoglobin. Increased NO may result in oxidative stress and damage to the fetus. During oxidative stress, ROS levels increase and GSH levels decrease [25,37]. Several teratogens affect developing embryos by increasing their oxidative stress through increased ROS levels, especially during early organogenesis. Oxidative stress, in turn, leads to severe embryo damage [38]. GSH is important in mouse embryo organogenesis, and GSH depletion significantly impacts oxidative stress and drug teratogenicity [39]. We examined GSH and ROS levels in immature oocytes and found that ROS levels increased following HU exposure, while GSH levels were decreased. These findings, which are consistent with previous studies of post-implantation embryos, suggest that the toxicological effects of HU extend to the maturation process. During embryonic development, cumulus expansion [31,40,41], apoptosis [41], and cell cycle regulation [42] are correlated with ROS levels in oocytes [43]. Paracrine factors regulate intracellular ROS levels during IVM of porcine oocytes [44]. In addition, regulation of cumulus cell lipid metabolism can increase GSH levels and decrease ROS levels during IVM, which results in improved developmental competence of somatic cell nuclear transfer embryos [45]. As GSH and ROS levels appear to be critical for IVM of oocytes, we also analyzed GSH and ROS levels of cumulus cells. Cumulus cells exposed to HU showed decreased GSH levels and increased ROS levels, which suggests that HU exposure led to oxidative stress in these cells. This oxidative stress may result in diminished signaling between cumulus cells and oocytes, and inhibition of oocyte maturation. The main endogenous source of ROS is mitochondria, where Δφm is a key regulator of mitochondrial respiration. Depolarization of Δφm can lead to excessive ROS production. Our results indicate that the oxidative stress of porcine oocytes and cumulus cells are synchronized, but the mechanism of HU-mediated oxidative stress in oocytes and cumulus cells is unclear and needs further study. Our current study investigated the role of mitochondria in HU toxicity and found that HU increased mitochondrial depolarization. Our findings indicate that HU may have an adverse effect on electron transfer in mitochondria that results in increased ROS.

The current study shows that the toxic effect of HU on porcine oocyte maturation *in vitro* is modulated through increased apoptosis of oocytes and cumulus cells, and increased oxidative stress. These effects hinder polar body extrusion in oocytes, which negatively influences their maturation and embryo development. The current study helps with risk assessment associated with HU exposure during oocyte maturation.

## Supplementary Material

Supplementary Table S1

## Data Availability

All data generated or used during the study are available from the corresponding author by request. All data generated or used during the study appear in the submitted article.

## Abbreviations

BSA: bovine serum albumin
CMF2HC: 4-chloromethyl-6,8-difluoro-7-hydroxycoumarin
COCs: cumulus–oocyte complexes
GSH: glutathione
HU: hydroxyurea
H2DCFDA: 2,7-dichlorodihydrofluorescein diacetate
IVC: *in vitro* culture
IVM: *in vitro* maturation
MII: metaphase II
PBS-PVA: phosphate-buffered saline mixed with 1g/l polyvinyl alcohol
PVA: polyvinyl alcohol
ROS: reactive oxygen species
RT-PCR: reverse transcription polymerase chain reaction

## References

[1] Brose R.D., Savonenko A., Devenney B., Smith K.D. and Reeves R.H. (2019) Hydroxyurea improves spatial memory and cognitive plasticity in mice and has a mild effect on these parameters in a down syndrome mouse model. Front. Aging Neurosci. 11, 96 10.3389/fnagi.2019.00096 31139073 PMC6527804

[2] Banh S. and Hales B.F. (2013) Hydroxyurea exposure triggers tissue-specific activation of p38 mitogen-activated protein kinase signaling and the DNA damage response in organogenesis-stage mouse embryos. Toxicol. Sci. 133, 298–308 10.1093/toxsci/kft069 23492809 PMC3663560

[3] Tohamy H.G., Gad El-Karim D.R. and El-Sayed Y.S. (2019) Attenuation potentials of royal jelly against hydroxyurea-induced infertility through inhibiting oxidation and release of pro-inflammatory cytokines in male rats. Environ. Sci. Pollut. Res. Int. 26, 21524–21534 10.1007/s11356-019-05521-3 31127524

[4] Woo G.H., Katayama K., Bak E.J., Ueno M., Yamauchi H., Uetsuka K. et al. (2004) Effects of prenatal hydroxyurea-treatment on mouse offspring. Exp. Toxicol. Pathol. 56, 1–7 10.1016/j.etp.2004.04.011 15581269

[5] Yan J. and Hales B.F. (2005) Activator protein-1 (AP-1) DNA binding activity is induced by hydroxyurea in organogenesis stage mouse embryos. Toxicol. Sci. 85, 1013–1023 10.1093/toxsci/kfi148 15772364

[6] Desesso J.M., Scialli A.R. and Goeringer G.C. (1994) D-mannitol, a specific hydroxyl free radical scavenger, reduces the developmental toxicity of hydroxyurea in rabbits. Teratology 49, 248–259 10.1002/tera.1420490404 8073363

[7] Hosako H., Little S.A., Barrier M. and Mirkes P.E. (2007) Teratogen-induced activation of p53 in early postimplantation mouse embryos. Toxicol. Sci. 95, 257–269 10.1093/toxsci/kfl143 17068108

[8] Larouche G. and Hales B.F. (2009) The impact of human superoxide dismutase 1 expression in a mouse model on the embryotoxicity of hydroxyurea. Birth Defects Res. A Clin. Mol. Teratol. 85, 800–807 10.1002/bdra.20595 19492401

[9] Chaube S. and Murphy M.L. (1966) The effects of hydroxyurea and related compounds on the rat fetus. Cancer Res. 26, 1448–1457 5911587

[10] Byrd D.C., Pitts S.R. and Alexander C.K. (1999) Hydroxyurea in two pregnant women with sickle cell anemia. Pharmacotherapy 19, 1459–1462 10.1592/phco.19.18.1459.30901 10600098

[11] Rodriguez-Vazquez L. and Marti J. (2018) An animal model for assessing the effects of hydroxyurea exposure suggests that the administration of this agent to pregnant women and young infants may not be as safe as we thought. Int. J. Mol. Sci. 19, 3986 10.3390/ijms19123986 30544930 PMC6320814

[12] Sampson M., Archibong A.E., Powell A., Strange B., Roberson S., Hills E.R. et al. (2010) Perturbation of the developmental potential of preimplantation mouse embryos by hydroxyurea. Int. J. Environ. Res. Public Health 7, 2033–2044 10.3390/ijerph7052033 20623009 PMC2898034

[13] Bjelica S., Diklic M., Dikic D., Kovacic M., Suboticki T., Mitrovic-Ajtic O. et al. (2019) Hydroxyurea-induced senescent peripheral blood mesenchymal stromal cells inhibit bystander cell proliferation of JAK2V617F-positive human erythroleukemia cells. FEBS J. 286, 3647–3663 10.1111/febs.14927 31090259

[14] Charton R., Muguet A., Griesenbeck J., Smerdon M.J. and Conconi A. (2019) In yeast cells arrested at the early S-phase by hydroxyurea, rRNA gene promoters and chromatin are poised for transcription while rRNA synthesis is compromised. Mutat. Res. 815, 20–29 10.1016/j.mrfmmm.2019.04.003 31063901

[15] Teng S., Ma C., Yu Y. and Yi C. (2019) Hydroxyurea promotes TET1 expression and induces apoptosis in osteosarcoma cells. Biosci. Rep. 39, BSR20190456 10.1042/BSR20190456 30988069 PMC6522705

[16] Bjelica S., Diklic M., Dikic D., Kovacic M., Suboticki T., Mitrovic-Ajtic O. et al. (2019) Hydroxyurea-induced senescent peripheral blood mesenchymal stromal cells inhibit bystander cell proliferation of JAK2V617F-positive human erythroleukemia cells. FEBS J. 286, 3647–3663 10.1111/febs.14927 31090259

[17] Kovacic P. (2011) Hydroxyurea (therapeutics and mechanism): metabolism, carbamoyl nitroso, nitroxyl, radicals, cell signaling and clinical applications. Med. Hypotheses 76, 24–31 10.1016/j.mehy.2010.08.023 20833482

[18] Perez-Pasten R., Martinez-Galero E. and Chamorro-Cevallos G. (2010) Quercetin and naringenin reduce abnormal development of mouse embryos produced by hydroxyurea. J. Pharm. Pharmacol. 62, 1003–1009 10.1111/j.2042-7158.2010.01118.x 20663034

[19] Yuan B., Liang S., Jin Y.X., Zhang M.J., Zhang J.B. and Kim N.H. (2017) Toxic effects of atrazine on porcine oocytes and possible mechanisms of action. PLoS ONE 12, e0179861 10.1371/journal.pone.0179861 28640859 PMC5480989

[20] Yuan B., Liang S., Jin Y.X., Kwon J.W., Zhang J.B. and Kim N.H. (2016) Progesterone influences cytoplasmic maturation in porcine oocytes developing in vitro. PeerJ 4, e2454 10.7717/peerj.2454 27672508 PMC5028735

[21] Zhang Y., Tan R., Wang L., Shi X., Li Y., Zhong X. et al. (2020) Shoutai pills improve the quality of oocytes exposed to the chemotherapeutic drug Hydroxyurea. Aging (Albany NY) 12, 8473–8483 10.18632/aging.103152 32388496 PMC7244078

[22] El Husseini N., Schlisser A.E. and Hales B.F. (2016) Editor’s highlight: hydroxyurea exposure activates the p53 signaling pathway in murine organogenesis-stage embryos. Toxicol. Sci. 152, 297–308 10.1093/toxsci/kfw089 27208086 PMC4960909

[23] Fang X., Yin H., Zhang H., Wu F., Liu Y., Fu Y. et al. (2019) p53 mediates hydroxyurea resistance in aneuploid cells of colon cancer. Exp. Cell. Res. 376, 39–48 10.1016/j.yexcr.2019.01.013 30684461

[24] Aboudehen K., Hilliard S., Saifudeen Z. and El-Dahr S.S. (2012) Mechanisms of p53 activation and physiological relevance in the developing kidney. Am. J. Physiol. Renal Physiol. 302, F928–F940 10.1152/ajprenal.00642.2011 22237799 PMC3330719

[25] Heo S.H., Cha Y. and Park K.S. (2014) Hydroxyurea induces a hypersensitive apoptotic response in mouse embryonic stem cells through p38-dependent acetylation of p53. Stem Cells Dev. 23, 2435–2442 10.1089/scd.2013.0608 24836177

[26] El Husseini N. and Hales B.F. (2018) The roles of P53 and its family proteins, P63 and P73, in the DNA damage stress response in organogenesis-stage mouse embryos. Toxicol. Sci. 162, 439–449 10.1093/toxsci/kfx270 29228353 PMC5888965

[27] Barbehenn E.K., Wales R.G. and Lowry O.H. (1974) The explanation for the blockade of glycolysis in early mouse embryos. Proc. Natl. Acad. Sci. U.S.A. 71, 1056–1060 10.1073/pnas.71.4.1056 4275392 PMC388161

[28] Eppig J.J. (1996) Coordination of nuclear and cytoplasmic oocyte maturation in eutherian mammals. Reprod. Fertil. Dev. 8, 485–489 10.1071/RD9960485 8870074

[29] Guo J., Shi L., Gong X., Jiang M., Yin Y., Zhang X. et al. (2016) Oocyte-dependent activation of MTOR in cumulus cells controls the development and survival of cumulus-oocyte complexes. J. Cell Sci. 129, 3091–3103 10.1242/jcs.182642 27358481 PMC5004896

[30] Budna J., Rybska M., Ciesiolka S., Bryja A., Borys S., Kranc W. et al. (2017) Expression of genes associated with BMP signaling pathway in porcine oocytes before and after IVM - a microarray approach. Reprod. Biol. Endocrinol. 15, 43 10.1186/s12958-017-0261-6 28576120 PMC5457624

[31] Dumesic D.A., Meldrum D.R., Katz-Jaffe M.G., Krisher R.L. and Schoolcraft W.B. (2015) Oocyte environment: follicular fluid and cumulus cells are critical for oocyte health. Fertil. Steril. 103, 303–316 10.1016/j.fertnstert.2014.11.015 25497448

[32] Russell D.L., Gilchrist R.B., Brown H.M. and Thompson J.G. (2016) Bidirectional communication between cumulus cells and the oocyte: Old hands and new players? Theriogenology 86, 62–68 10.1016/j.theriogenology.2016.04.019 27160446

[33] Tanghe S., Van Soom A., Nauwynck H., Coryn M. and de Kruif A. (2002) Minireview: Functions of the cumulus oophorus during oocyte maturation, ovulation, and fertilization. Mol. Reprod. Dev. 61, 414–424 10.1002/mrd.10102 11835587

[34] Park H.J., Chae S.K., Kim J.W., Yang S.G., Jung J.M., Kim M.J. et al. (2017) Ganglioside GM3 induces cumulus cell apoptosis through inhibition of epidermal growth factor receptor-mediated PI3K/AKT signaling pathways during in vitro maturation of pig oocytes. Mol. Reprod. Dev. 84, 702–711 10.1002/mrd.22848 28585705

[35] Villa-Diaz L.G. and Miyano T. (2004) Activation of p38 MAPK during porcine oocyte maturation. Biol. Reprod. 71, 691–696 10.1095/biolreprod.103.026310 15115730

[36] Shimada M., Ito J., Yamashita Y., Okazaki T. and Isobe N. (2003) Phosphatidylinositol 3-kinase in cumulus cells is responsible for both suppression of spontaneous maturation and induction of gonadotropin-stimulated maturation of porcine oocytes. J. Endocrinol. 179, 25–34 10.1677/joe.0.1790025 14529562

[37] Nader E., Grau M., Fort R., Collins B., Cannas G., Gauthier A. et al. (2018) Hydroxyurea therapy modulates sickle cell anemia red blood cell physiology: Impact on RBC deformability, oxidative stress, nitrite levels and nitric oxide synthase signalling pathway. Nitric Oxide 81, 28–35 10.1016/j.niox.2018.10.003 30342855

[38] Ornoy A. (2007) Embryonic oxidative stress as a mechanism of teratogenesis with special emphasis on diabetic embryopathy. Reprod. Toxicol. 24, 31–41 10.1016/j.reprotox.2007.04.004 17548185

[39] Yan J. and Hales B.F. (2006) Depletion of glutathione induces 4-hydroxynonenal protein adducts and hydroxyurea teratogenicity in the organogenesis stage mouse embryo. J. Pharmacol. Exp. Ther. 319, 613–621 10.1124/jpet.106.109850 16902051

[40] Su Y.Q., Sugiura K. and Eppig J.J. (2009) Mouse oocyte control of granulosa cell development and function: paracrine regulation of cumulus cell metabolism. Semin. Reprod. Med. 27, 32–42 10.1055/s-0028-1108008 19197803 PMC2742468

[41] Fu X.H., Chen C.Z., Wang Y., Peng Y.X., Wang W.H., Yuan B. et al. (2019) COL1A1 affects apoptosis by regulating oxidative stress and autophagy in bovine cumulus cells. Theriogenology 139, 81–89 10.1016/j.theriogenology.2019.07.024 31377650

[42] Fu X.H., Chen C.Z., Li S., Han D.X., Wang Y.J., Yuan B. et al. (2019) Dual-specificity phosphatase 1 regulates cell cycle progression and apoptosis in cumulus cells by affecting mitochondrial function, oxidative stress, and autophagy. Am. J. Physiol. Cell Physiol. 317, C1183–C1193 10.1152/ajpcell.00012.2019 31532716

[43] El Sheikh M., Mesalam A., Mesalam A.A., Idrees M., Lee K.L. and Kong I.K. (2019) Melatonin abrogates the anti-developmental effect of the AKT inhibitor SH6 in bovine oocytes and embryos. Int. J. Mol. Sci. 20, 2956 10.3390/ijms20122956 PMC6627520 31212969

[44] Yoon J.D., Hwang S.U., Kim E., Jin M., Kim S. and Hyun S.H. (2017) GDF8 activates p38 MAPK signaling during porcine oocyte maturation in vitro. Theriogenology 101, 123–134 10.1016/j.theriogenology.2017.06.003 28708509

[45] Lee S., Jin J.X., Khoirinaya C., Kim G.A. and Lee B.C. (2016) Lanosterol influences cytoplasmic maturation of pig oocytes in vitro and improves preimplantation development of cloned embryos. Theriogenology 85, 575–584 10.1016/j.theriogenology.2015.09.041 26494176

