# Supplementary material for: Hydroxyurea affects in vitro porcine oocyte maturation through increased apoptosis and oxidative stress
Source: Biosci Rep. 2021 Apr 22;41(4):BSR20203091. doi: 10.1042/BSR20203091 (PMC8062957; doi:10.1042/BSR20203091)
Supplement: Supplementary Table S1 [file BSR-2020-3091_supp.pdf]

Supplementary Table 1. Primer sequences used for real-time PCR

Gene	Reference	Primer	Sequence	Annealing	Size
P53	AF098067	Forward	CCCCAGCATCTCATCCGCAA	61 °C	254bp
		Reverse	ACACGCACCTCAAAGC		
Caspase-3	NM_214131.1	Forward	TTTGCGTGCTTCTAAGCCAT	60 °C	147bp
		Reverse	GGCAGGCCTGAATTATGAAA		
β-actin	U07786	Forward	GTGGACATCAGGAAGGACCTCTAA	61 °C	137bp
		Reverse	TGATCTTGATCTTCATGGTGCT		
